# Combined chemotherapy with sequential allogeneic hematopoietic stem cell transplantation successfully treats primary plasma cell leukemia: a case report

**DOI:** 10.3389/fonc.2026.1842869

**Published:** 2026-05-22

**Authors:** Xiushuai Dong, Jinghua Wang, Yaoyao Tian, Lianjie Wang, Wei Wang

**Affiliations:** Department of Hematology, The Second Affiliated Hospital, Harbin Medical University, Harbin, Heilongjiang, China

**Keywords:** allogeneic hematopoietic stem cell transplantation, case report, chemotherapy, long-term survival, primary plasma cell leukemia

## Abstract

Primary plasma cell leukemia (pPCL) is an extremely aggressive and rare subtype of plasma cell neoplasm with extremely poor prognosis, and optimal treatment strategies remain controversial due to its low incidence. This case is unique in that it reports long-term disease-free survival after combination chemotherapy followed by allogeneic peripheral blood hematopoietic stem cell transplantation (allo-PBSCT), which provides valuable real-world evidence supporting the potential curative role of this sequential therapeutic approach for pPCL, a disease where long-term survival is rarely achieved with conventional treatments.

## Introduction

Primary plasma cell leukemia (pPCL) is a rare and highly malignant plasma cell tumor characterized by anemia, infection, bleeding and extramedullary invasion. pPCL is characterized by an acute onset, short disease course, poor prognosis, and a median overall survival of less than 1 year (1). Historically, the International Myeloma Working Group (IMWG) required ≥20% circulating plasma cells (CPCs) to diagnose pPCL, but accumulating evidence shows that the prognosis of patients with 5–19% CPCs is comparable to that of patients with classical pPCL, a discovery that prompted the IMWG’s 2021 update to the diagnostic criterion, lowering it to ≥5% CPCs (2). For the treatment of pPCL, the IMWG recommends an intense chemotherapy followed by hematopoietic stem cell transplantation with a bortezomib-based regimen (3). However, due to the rarity of pPCL, the timing of transplantation and the efficacy of autologous or allo-HSCT remain under debate. In this study, we intend to report a case of pPCL treated with allo-HSCT after chemotherapy.

## Case description

A 47-year-old female patient presented to the Otolaryngology Department of our hospital in May 2017 with dyspnea, and laryngeal examination revealed glottic masses. In July 2017, the initial tracheotomy was performed because of severe dyspnea at a hospital in Beijing. One piece of subglottic mass was surgically resected. The specimen was grayish-red in appearance, with a size of 0.7 × 0.5 × 0.3 cm. Pathological examination confirmed a plasma cell neoplasm. Immunohistochemistry of the subglottic mass indicated that CD38, CD138, BCL2, Lambda and ki-67 (30%) were positive, and Kappa, CD3, CD20, CD34, CD56, CK, LCA, Syn were negative. Bone marrow puncture indicated grade III hyperplasia, with abnormal plasma cells accounting for 38.8%. Abnormal plasma cells in peripheral blood were detected by flow cytometry, accounting for 24%. According to the IMWG diagnostic criteria at that time, the patient was diagnosed with pPCL complicated with extramedullary plasmacytoma. Given the poor prognosis and short survival of pPCL, the patient refused anti-tumor treatment and was discharged against medical advice. On September 4, 2017, she was admitted to our department with obvious aggravation of dyspnea for symptomatic treatment and symptom relief.

Admission physical examination: the general condition was extremely poor, in a critical state, with mild anemia; tracheotomy and a tracheotrocar were seen in the neck. Laboratory examination: routine blood test showed white blood cells 12.8×10^9^/L, hemoglobin 94 g/L, platelet 187×10^9^/L, and 20% of plasma cells were found on peripheral blood smear (absolute value: 2.56×10^9^/L). Biochemical results showed creatinine 54 μmol/L, calcium ion 2.19 mmol/L, total protein 67.3 g/L, albumin 41.1 g/L, globulin 26.2 g/L, lactate dehydrogenase 145 U/L. Immunoglobulin tests showed IgG: 3.76 ↓(reference range: 7.0-16.0 g/L), IgA: < 0.261 ↓(reference range: 0.7-4.0 g/L), IgM: 0.19 g/L ↓(reference range: 0.4-2.3), IgE: < 19 (reference range: 0-100.0 Iu/mL); light chain tests showed Kappa: 0.78 ↓(reference range: 1.7-3.7 g/L), Lambda: 2.77 ↑(reference range: 0.9-2.1 g/L), K/L: 0.28; urine light chain tests showed Kappa: 28.3↑,(reference range: 0-7.1 mg/L),Lambda: 2670↑ (reference range: 0-3.9 mg/L), K/L: 0.01; blood β2 microglobulin: 2799 μg/L ↑. Blood and urine immunofixation electrophoresis showed visible Lambda monoclonal bands. Bone marrow puncture indicated grade III hyperplasia, with 68.5% abnormal plasma cells and 20% peripheral plasma cells. The patient refused flow cytometry, chromosome and FISH tests. Skull anteroposterior and lateral radiographs suggested reduced localized density in the posterior skull. No abnormality was found on pelvic X-ray, and no abnormalities were found on ECG or chest CT. The patient had no significant past medical history, no family history of hematological malignancies or other hereditary diseases. The patient was diagnosed as pPCL with extramedullary infiltration, and had undergone tracheotomy and placement of a tracheotrocar.

Bortezomib-based chemotherapy followed by transplantation was recommended, but the patient refused the regimen for economic reasons. Ultimately, she reluctantly consented to DCEP+T chemotherapy, the regimen details were as follows: dexamethasone 40 mg for 4 days, cyclophosphamide 0.4 g for 4 days, etoposide 0.1 g for 4 days, cisplatin 40 mg for 4 days, thalidomide 75 mg orally every night. The main adverse effects were nausea and vomiting, with no significant hematological toxicities. After the first course of treatment, the patient had no dyspnea and her condition improved significantly. Therefore, DCEP+T regimen chemotherapy was continued, with partial remission (PR) achieved after 2 courses and complete remission (CR) achieved after 3 courses (the patient refused flow cytometry examination). Transplantation was then recommended for the patient. While awaiting transplantation, the patient received an additional course of DCEP+T and one course of DCEP+R (lenalidomide 10 mg, days 1–21) sequentially.

In April 2018, she received allogeneic peripheral blood stem cell transplantation (allo-HSCT). The donor was the patient’s sister, with 7/10 HLA matching compatibility, and both the patient and the donor had B+ blood type. The apheresis volume was 310 ml, with white blood cells 251×10^9^/L, mononuclear cells accounting for 91%. The patient’s body weight was 50 kg, with MNC 14.1×10^8^/kg and CD34 9.45×10^6^/kg. The pretransplant conditioning regimen was fludarabine combined with melphalan: fludarabine (25 mg/m² for 5 days, days -10 to -6), melphalan (200 mg/m² for 2 days, days -5 to -4), ATG (Rabbit Anti-human Thymocyte Immunoglobulin, Sanofi Winthrop Industrie, 125 mg for 4 days, days -5 to -2), bortezomib (1.3 mg/m² for 2 days, days -9 and -2), semustine 350 mg on day -3. Graft-versus-host disease (GVHD) prophylaxis consisted of cyclosporine, methotrexate (MTX), and mycophenolate mofetil.

One month after transplantation, bone marrow flow cytometry revealed only 0.003% normal plasma cells, and complete donor chimerism was 99.91%; the patient received no maintenance therapy. The patient discontinued regular follow-up at 7 months post-transplant, and immunosuppressive agents were withdrawn. Complete donor chimerism was 99.33% at 18 months post-transplant. At present, 8 years after transplantation, the patient remains in normal clinical condition with regular follow-up.

## Discussion

pPCL is a rare, highly aggressive plasma cell malignancy that presents as leukemic phase without a prior history of multiple myeloma (MM), and accounts for 50–70% of all plasma cell leukemia (PCL) cases. The prognosis is poor, and it is often accompanied by extramedullary infiltration. The median age at onset of pPCL is 52–65 years, with clinical manifestations including anemia, bleeding, infection, hypercalcemia, renal insufficiency, and extramedullary plasmacytoma. The incidence of osteolytic lesions in pPCL is lower than that in MM; the proportion of light chain subtype is 26–44%, which is higher than that in MM, and IgA subtype is rare in pPCL. The typical immunophenotypes of pPCL are positive for CD38, CD138, CD20, CD45, CD19 and CD123, with low expression of CD27; CD71, CD117 and HLA-DR are less expressed. The decreased expression of CD56 may be related to the reduced osteolysis ability and increased extramedullary migration of tumor cells, and the high expression of CD44 may be involved in cell adhesion and migration. Cytogenetic analyses of pPCL cases mainly show a hypodiploid karyotype, with IgH translocation mainly t (11; 14); there is no significant statistical difference in the incidence of 13q deletion between pPCL and sPCL, and the detection rate of 17p deletion is approximately 35%.Furthermore, a subset of pPCL patients with 5%–19% circulating plasma cells may exhibit an occult clinical presentation, mild symptoms, and normal LDH levels (2). Some affected individuals are even entirely asymptomatic and diagnosed incidentally during routine laboratory examination. Normal LDH likely represents a characteristic feature of occult or indolent pPCL (4). Nevertheless, this entity remains associated with a high-risk prognosis despite its indolent initial presentation.

The main therapeutic goals for pPCL are to prolong overall survival and improve patients’ quality of life, and the optimal induction regimen remains unknown. In 2013, the IMWG issued treatment recommendations for newly diagnosed pPCL patients (3): For transplant-eligible patients, a bortezomib-based combination chemotherapy regimen is recommended; patients aged < 50 years may be considered for myeloablative allo-HSCT; for patients over 50 years old, autologous hematopoietic stem cell transplantation is recommended, and in those with an available donor, reduced-intensity conditioning (RIC) allogeneic transplantation is recommended. In the absence of an available donor, consolidation or maintenance therapy based on bortezomib plus or minus lenalidomide may be considered. For transplant-ineligible patients, bortezomib-based induction chemotherapy is the first-line option; continuous maintenance therapy is recommended for patients who achieve a partial response (PR) or better, and patients with insufficient therapeutic response may be enrolled in clinical trials or receive palliative care.

However, there is limited transplant-related data for pPCL, and most relevant literature consists of retrospective studies, with well-designed prospective studies being scarce. Literature reports on the efficacy of allogeneic versus autologous transplantation in pPCL are inconsistent, and the optimal timing of transplantation remains undefined. Lawless et al. demonstrated that tandem transplantation represents the optimal strategy for pPCL. Patients not achieving complete remission (CR) benefit most from autologous-allogeneic tandem transplantation, while those achieving CR are best suited for tandem autologous transplantation. Upfront allogeneic transplantation is associated with a lower relapse rate but extremely high non-relapse mortality (NRM), and is therefore not recommended as first-line transplantation (5). Reduced-intensity conditioning (RIC) allogeneic transplantation has insufficient anti-PCL activity. The recurrence rate is high in the autologous transplantation group (6).

The European Myeloma Network (EMN) has issued a review and consensus statement on pPCL, which is based on the 2021 IMWG revised diagnostic criteria (≥5% clonal circulating plasma cells in peripheral blood) and defines stratified treatment regimens for transplant-eligible, transplant-ineligible, and relapsed/refractory patients. Musto et al. stated that the standard treatment workflow for transplant-eligible PPCL patients is as follows: first-line induction with 4 cycles of a four-drug regimen containing an anti-CD38 monoclonal antibody, followed by tandem autologous stem cell transplantation, consolidation with 2–4 cycles of the same regimen, and long-term maintenance with lenalidomide plus an anti-CD38 antibody or a proteasome inhibitor (PI). For patients who do not achieve complete remission (CR) after induction, sequential reduced-intensity conditioning allogeneic stem cell transplantation following autologous transplantation may be considered (7).

At a time when proteasome inhibitors and anti-CD38 monoclonal antibodies were unavailable, this patient initially received a five-drug combination chemotherapy regimen. After achieving complete remission (CR), the patient underwent allogeneic hematopoietic stem cell transplantation and was successfully treated. The total cost of five cycles of induction chemotherapy before transplantation was 150,000 Chinese Yuan (CNY), and the cost of transplantation was an additional 230,000 CNY. To date, the patient has remained in CR with uneventful follow-up for nearly 8 years post-transplant. The patient emphasizes that while the treatment regimen is physically demanding, the resulting 8-year disease-free survival and restored quality of life make the experience worthwhile, highlighting the profound personal benefit of curative-intent transplantation for this aggressive disease. Based on the successful therapeutic experience of this case, our center proposes that the intensity of cytotoxic chemotherapy should be increased during pPCL induction therapy, such as a 4–5 drug combination regimen incorporating novel agents. Young patients should achieve CR as early as possible and undergo myeloablative allo-HSCT promptly. However, due to the rarity of pPCL, well-designed clinical trials are lacking. However, the absence of cytogenetic and FISH data at diagnosis hinders the interpretation of the clinical outcomes. Furthermore, the patient had poor compliance in the early phase of treatment and did not receive post-transplant maintenance therapy, yet still achieved durable complete remission CR. Whether maintenance therapy is warranted after allogeneic transplantation, as well as the optimal maintenance regimen, remains unclear.

In contrast to secondary plasma cell leukemia (sPCL), HSCT demonstrates profound clinical value and prognostic impact in pPCL. Accordingly, HSCT is strongly recommended as a frontline priority intervention for all transplant-eligible pPCL patients (8). Auto-HSCT is associated with a favorable safety profile (NRM 5%–7%) and a 3-year OS of 51%, but is hampered by a high relapse rate of 68%. Allo-HSCT reduces relapse through the graft-versus-leukemia effect, but entails higher treatment-related mortality (12%) and increased risks of acute GVHD (27%) and chronic GVHD (36%), leading to greater early mortality (9). Notably, in the novel agent era, induction with novel drugs such as bortezomib and achievement of CR or very good partial response (VGPR) before transplantation may improve survival outcomes among transplant-eligible pPCL patients. Well-designed and comprehensive clinical trials are therefore urgently warranted to overcome the dismal prognosis of pPCL.

## Conclusion

This case report demonstrates that combination chemotherapy followed by peripheral blood allo-HSCT can achieve durable long-term survival in pPCL, with the patient remaining disease-free for nearly 8 years. Given the aggressive nature and historically poor prognosis of pPCL, this outcome supports allo-PBSCT as a curative-intent strategy for eligible patients who achieve complete remission with induction chemotherapy. Further prospective studies are needed to optimize induction regimens, donor selection, and post-transplant care to generalize these findings and improve outcomes for this rare hematologic malignancy.

Despite the favorable long-term outcome, this single case report has inherent limitations, including the lack of a control group and the inability to generalize findings to all pPCL patients, especially those unfit for transplantation due to age or comorbidities. Additionally, the optimal timing of transplantation post-remission and the role of post-transplant maintenance therapy remain unclear. Future research should focus on prospective clinical trials to evaluate the efficacy of novel induction regimens combined with allo-PBSCT, identify predictive biomarkers for transplant success, and develop targeted strategies to reduce transplant-related mortality and relapse risk in pPCL.

## Data Availability

The data analyzed in this study is subject to the following licenses/restrictions: The dataset contains sensitive patient clinical information and is protected by medical privacy regulations and institutional ethics requirements. Full access is restricted to protect patient confidentiality. De-identified, limited data may be provided by the corresponding author upon reasonable academic request, with ethics committee approval. Requests to access these datasets should be directed to Xiushuai Dong, dongxiushuai@163.com.

## References

[1] Avet-Loiseau H Roussel M Campion L Leleu X Marit G Jardel H . Cytogenetic and therapeutic characterization of primary plasma cell leukemia: the IFM experience. Leukemia. (2012) 26:158–9. doi: 10.1038/leu.2011.176. PMID: 21799511

[2] Fernández de Larrea C Kyle R Rosiñol L Paiva B Engelhardt M Usmani S . Primary plasma cell leukemia: consensus definition by the International Myeloma Working Group according to peripheral blood plasma cell percentage. Blood Cancer J. (2021) 11:192. doi: 10.1038/s41408-021-00587-0. PMID: 34857730 PMC8640034

[3] International Myeloma Working Group. Plasma cell leukemia: consensus statement on diagnostic requirements, response criteria and treatment recommendations by the International Myeloma Working Group. Leukemia. (2013) 27:780–91. doi: 10.1038/leu.2012.336. PMID: 23288300 PMC4112539

[4] Nandakumar B Kumar SK Dispenzieri A Buadi FK Dingli D Lacy MQ . Clinical characteristics and outcomes of patients with primary plasma cell leukemia in the era of novel agent therapy. Mayo Clin Proc. (2021) 96:677–87. doi: 10.1016/j.mayocp.2020.06.060. PMID: 33673918 PMC7939118

[5] Lawless S Iacobelli S Knopova NS Chevallier P Blaise D Milpied N . Comparison of autologous and allogeneic hematopoietic cell transplantation strategies in patients with primary plasma cell leukemia, with dynamic prediction modeling. Haematologica. (2023) 108:1105–14. doi: 10.3324/haematol.2021.280568. PMID: 35770529 PMC10071135

[6] Mahindra A Kalaycio ME Vela-Ojeda J Vesole DH Zhang MJ Li P . Hematopoietic cell transplantation for primary plasma cell leukemia: results from the Center for International Blood and Marrow Transplant Research. Leukemia. (2012) 26:1091–7. doi: 10.1038/leu.2011.312. PMID: 22042147 PMC3274611

[7] Musto P Engelhardt M van de Donk NWCJ Gay F Terpos E Einsele H . European Myeloma Network Group review and consensus statement on primary plasma cell leukemia. Ann Oncol. (2025) 36:556–72. doi: 10.1016/j.annonc.2025.01.022. PMID: 39924085

[8] Shalaby K Azad F Parker S Wang C Yu H Attwood K . Clinical characteristics and survival outcomes of patients with primary and secondary plasma cell leukemia according to the 2021 definition: A single center retrospective study. Clin Lymphoma Myeloma Leuk. (2025) 25:67–75. doi: 10.1016/j.clml.2024.10.014. PMID: 39578203

[9] Shahzad M Iqbal Q Amin MK Irfan S Warraich SZ Anwar I . Outcomes of hematopoietic stem cell transplantation in primary plasma cell leukemia: a systematic review and meta-analysis. Leuk Res. (2025) 148:107640. doi: 10.1016/j.leukres.2024.107640. PMID: 39724831

